# Supplementary material for: Human intestinal organoid-derived PDGFRα + mesenchymal stroma enables proliferation and maintenance of LGR4 + epithelial stem cells
Source: Stem Cell Res Ther. 2024 Jan 17;15:16. doi: 10.1186/s13287-023-03629-5 (PMC10792855; doi:10.1186/s13287-023-03629-5)
Supplement: Supplementary file 1 — Additional file1: Generation of intestinal epithelial and mesenchymal stromal cells from intestinal organoids (A) Experimental scheme for generation of the intestinal epithelial and mesenchymal stromal cells from small intestinal organoids. Intestinal epithelial cells and mesenchymal stromal cells were isolated at 21 and 28 days, respectively. (B) Details of intestinal epithelial and mesenchymal stromal cells established from human iPSC- and ESC-derived intestinal organoids. [file 13287_2023_3629_MOESM1_ESM.pdf]

A

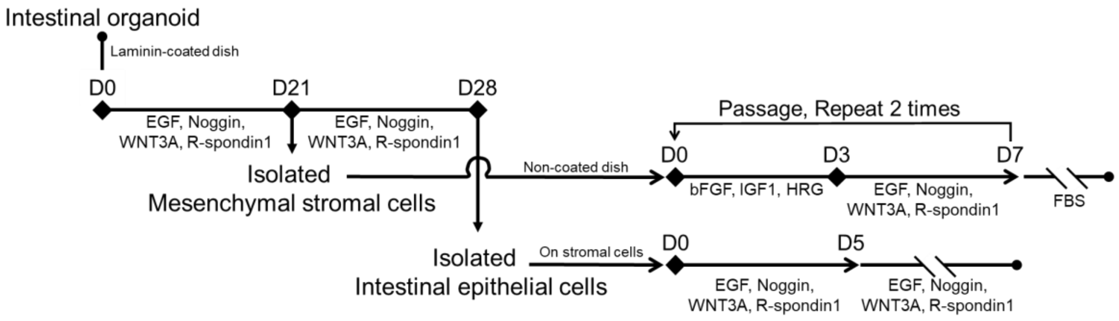

B

Organoid Number	Origin	RYU Number	Established	LONG Number	Established	Condition
Intestinal organoid #1	Human iPSCs	RYU01	Success	LONG01	Success	RYU：Normal , LONG：Normal
Intestinal organoid #2	Human ESCs	RYU02	Success	LONG02	Success	RYU：Normal , LONG：Normal
		RYU03	Success	LONG03	Success	RYU：Normal , LONG：Normal
Intestinal organoid #3	Human ESCs	RYU04	Success	LONG04	Success	RYU：Normal , LONG：Normal
Intestinal organoid #4	Human ESCs	RYU05	Success	LONG05	Success	RYU：Normal , LONG：Normal
Intestinal organoid #5	Human iPSCs	RYU06	Failure	LONG06	Success	RYU：Proliferation of non-intestinal cells , LONG：Normal
Intestinal organoid #6	Human iPSCs	RYU07	Success	LONG07	Success	RYU：Normal , LONG：Normal
Intestinal organoid #7	Human iPSCs	RYU08	Failure	LONG08	Success	RYU：Non-proliferative , LONG：Normal

Figure S1. Generation of intestinal epithelial and mesenchymal stromal cells from intestinal organoids
