# Supplementary figures and images for: Human intestinal organoid-derived PDGFRα + mesenchymal stroma enables proliferation and maintenance of LGR4 + epithelial stem cells
Source: Stem Cell Res Ther. 2024 Jan 17;15:16. doi: 10.1186/s13287-023-03629-5 (PMC10792855; doi:10.1186/s13287-023-03629-5)

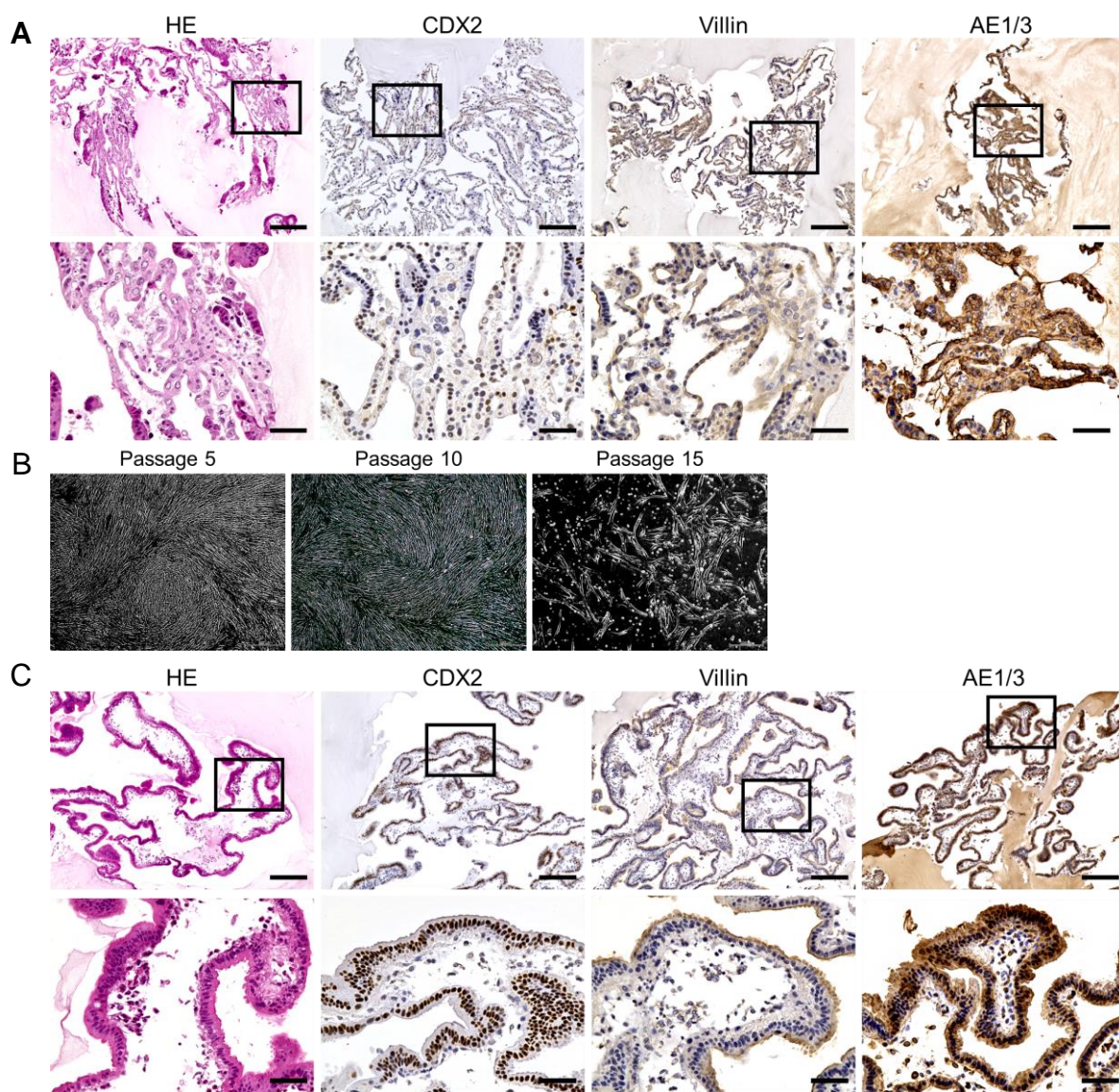

**Figure S2. Mesenchymal stromal cells generated intestinal epithelial cells**

Supplement: Supplementary file 2 — Additional file2: Mesenchymal stromal cells supported intestinal epithelial cells (A) Histology and immunohistochemistry of RYU on MEFs. Immunohistochemistry was performed with antibodies to CDX2, Villin, and AE1/AE3. Scale bars: 200 µm (upper panels), 50 µm (lower panels). (B) Phase-contrast photomicrographs of LONG at Passages 5, 10, and 15. LONG ceased their proliferation at Passage 15. Scale bars: 500 µm. (C) Histology and immunohistochemistry of RYU on LONG. Scale bars: 200 µm (upper panels), 50 µm (lower panels). [file 13287_2023_3629_MOESM2_ESM.pdf]

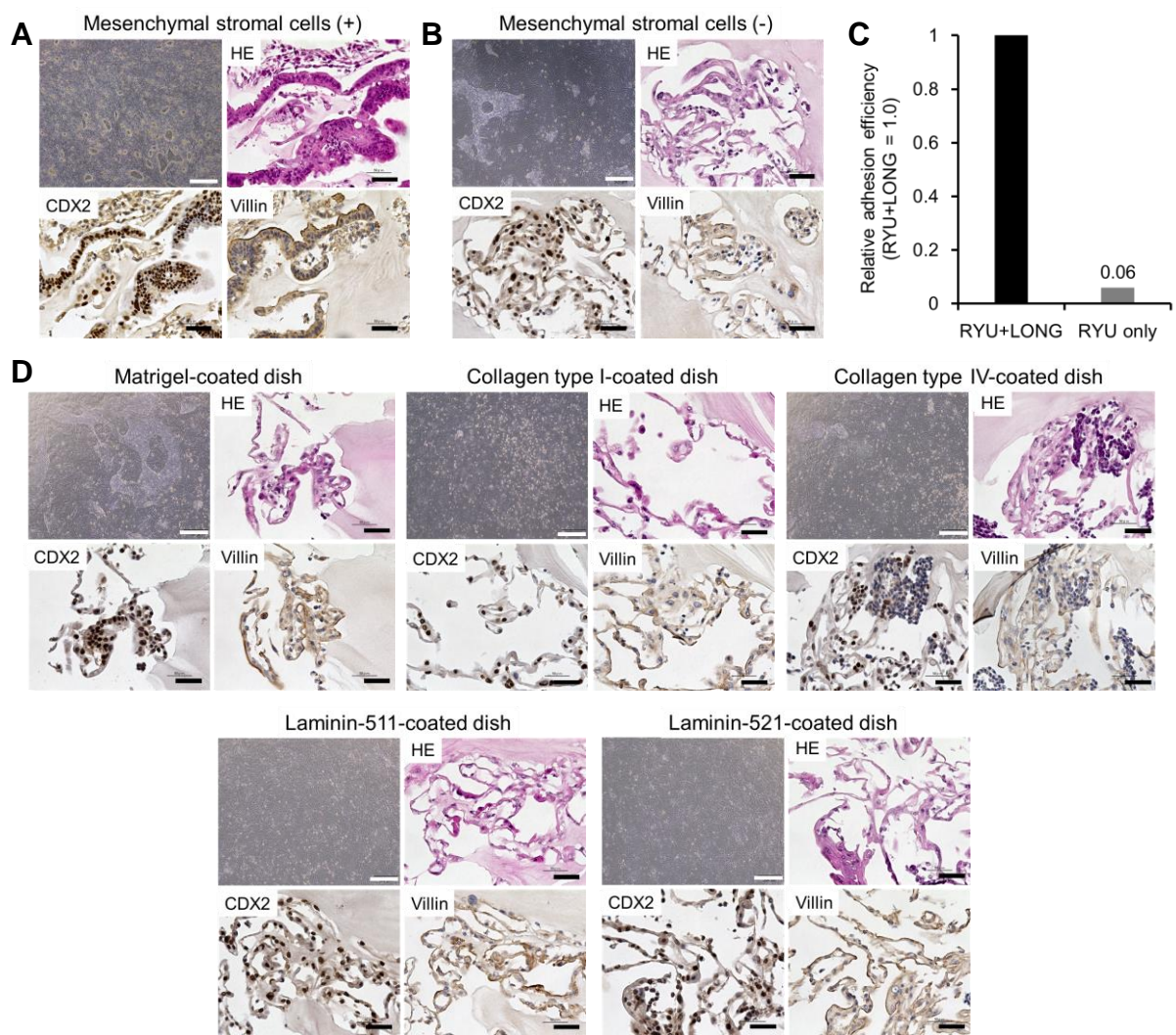

**Figure S3. Intestinal epithelial cells require mesenchymal stromal cells**

Supplement: Supplementary file 3 — Additional file3: Intestinal epithelial cells require mesenchymal stromal cells (A) Phase-contrast photomicrograph (left-upper) and histology (the others) of RYU on RYU. (Right-upper) H.E. stain. (Lower) Immunohistochemistry of RYU with antibodies to CDX2 (left-lower) and Villin (right-lower). Scale bars: 500 µm (left-upper) and 50 µm (the others). (B) Phase-contrast photomicrograph (left-upper) and histology (the others) of RYU on the non-coated dish. (Right-upper) H.E. stain. (Lower) Immunohistochemistry of RYU with antibodies to CDX2 (left-lower) and Villin (right-lower). Scale bars: 500 µm (left-upper) and 50 µm (the others). (C) Relative adhesion efficiency of RYU on LONG or no feeder. The number of colonies for RYU cultured on LONG was set to 1.0. RYU cultured on LONG resulted in 392 colonies. RYU cultured on no feeder yielded 23 colonies. (D) Phase-contrast photomicrograph (left-upper) and histology (the others) of RYU on the extracellular matrix-coated dish (Matrigel, Collagen type-I, IV, Laminin-511 and 521). (Right-upper) H.E. stain. (Lower) Immunohistochemistry of RYU with antibodies to CDX2 (left-lower) and Villin (right-lower). Scale bars: 500 µm (left-upper) and 50 µm (the others). [file 13287_2023_3629_MOESM3_ESM.pdf]

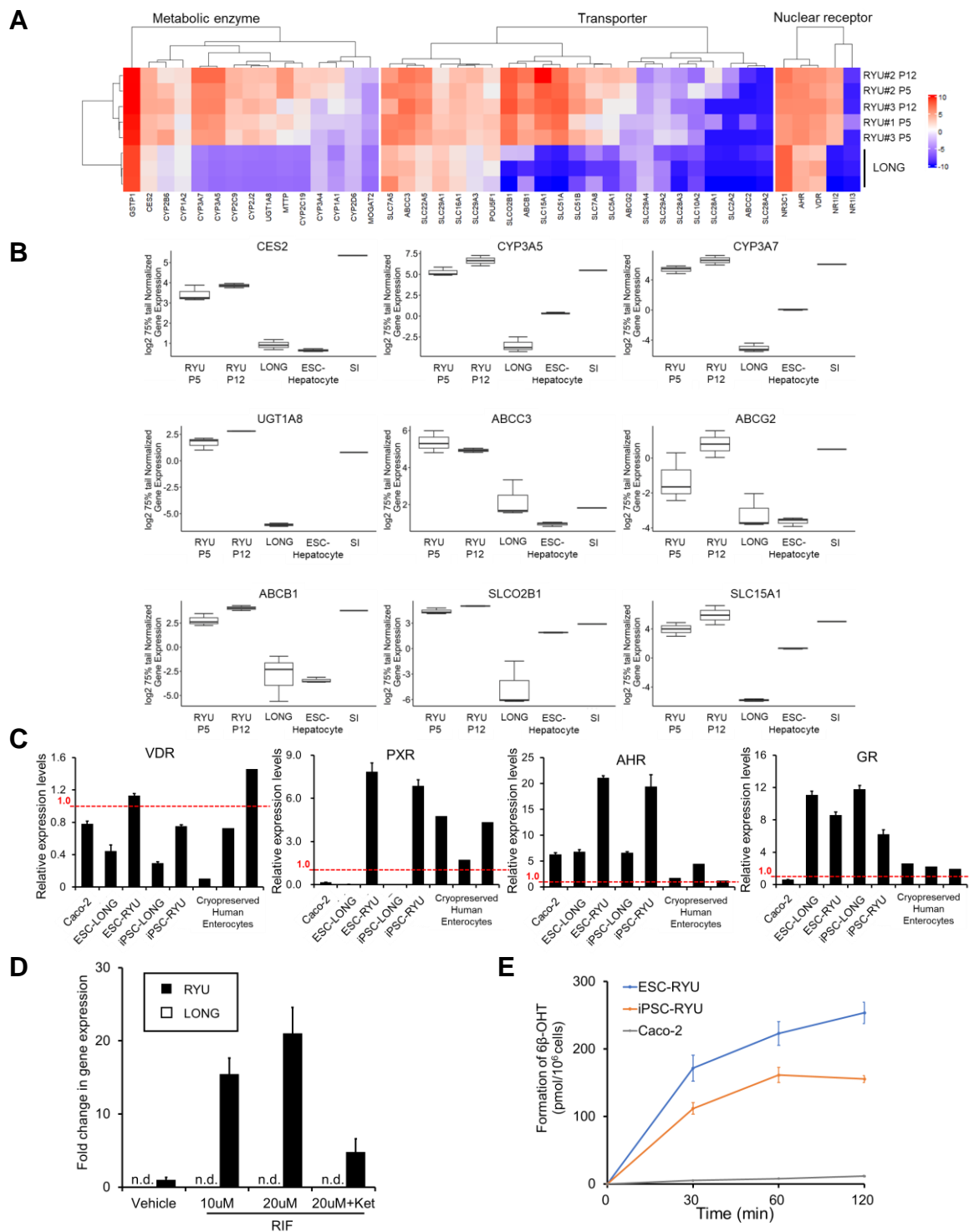

Figure S6. Pharmacokinetics-related gene expression in intestinal epithelial cells

Supplement: Supplementary file 6 — Additional file6: Pharmacokinetics-related gene expression in intestinal epithelial cells (A) Heat map of gene expression in RYU (Passages 5 and 12) and LONG. Expression levels were calculated from the results of triplicate biological experiments. (B) Boxplot of representative human intestinal epithelial stem cells and differentiated cell markers. Boxplots are expressed as mean ± SD (n = 3 triplicate biological experiments). (C) Expression of the genes for nuclear receptors (VDR, PXR, AHR, and GR). The expression level of adult small intestine whole tissue was set to 1.0. Expression levels were calculated from the results of triplicate biological experiments. Results are expressed as mean ± SD (n = 3 triplicate biological experiments). ES-LONG: human ESC-derived mesenchymal stromal cells, ES-RYU: human ESC-derived intestinal epithelial cells, iPS-LONG: human iPSC-derived mesenchymal stromal cells, iPS-RYU: human iPSC-derived intestinal epithelial cells. (D) Inhibition and induction of the cytochrome P450 genes with exposure to Rifampicin (Rif) and Ketoconazole (Ket). Results are expressed as mean ± SD (n = 3). The expression level of each gene without any treatment (DMSO) was set to 1.0. Expression levels were calculated from the results of independent (biological) triplicate experiments. (E) 6β-hydroxy testosterone concentration over time in CYP3A4 activity tests. ES-RYU: human ESC-derived intestinal epithelial cells (blue), iPS-RYU: human iPSC-derived intestinal epithelial cells (orange), Caco-2 (gray). [file 13287_2023_3629_MOESM6_ESM.pdf]

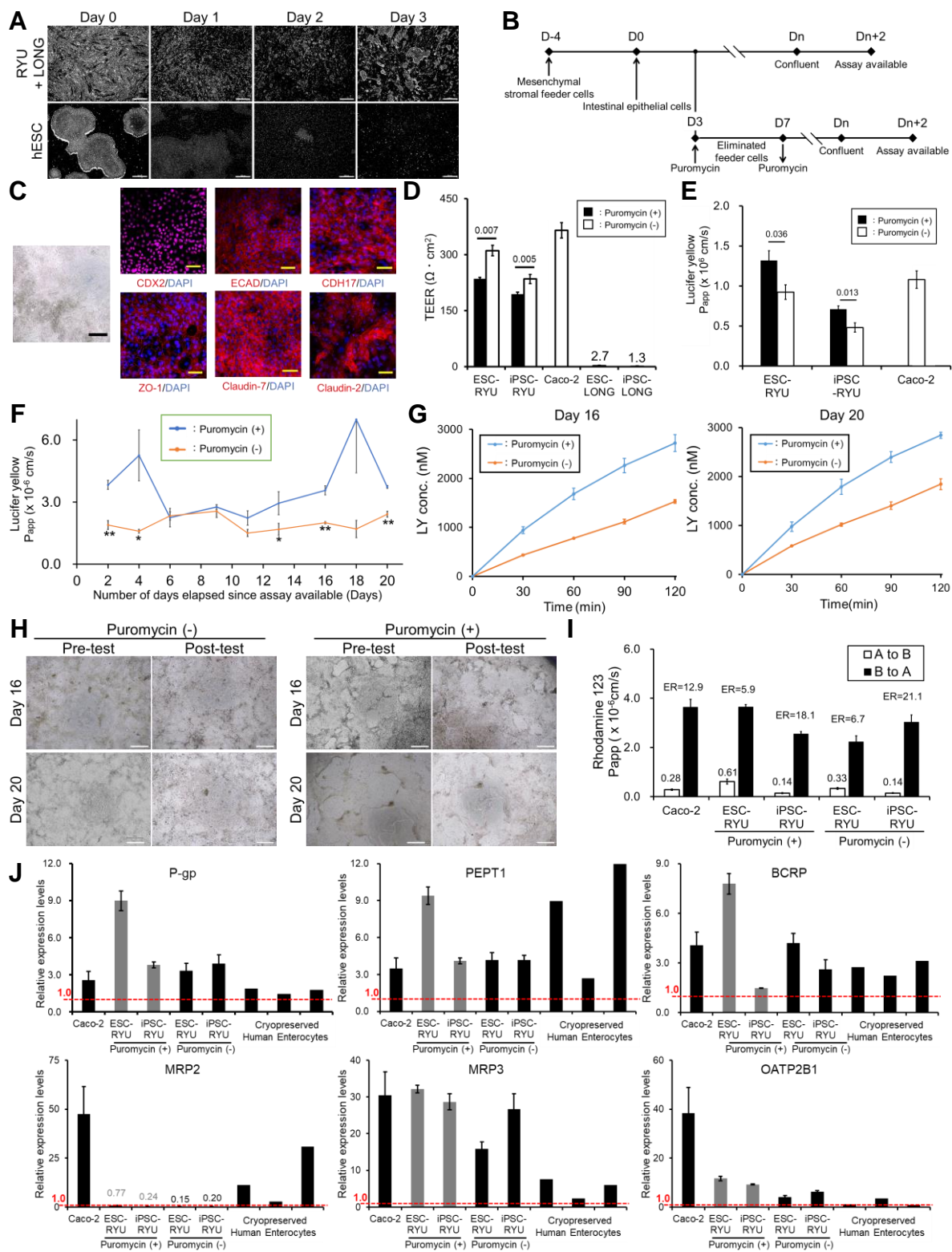

**Figure S7. Puromycin-based selection of intestinal epithelial cells**

Supplement: Supplementary file 7 — Additional file7: Puromycin-based selection of intestinal epithelial cells (A) Phase-contrast photomicrographs of RYU, LONG and human ESCs upon exposure to puromycin. Puromycin (1 µg/mL) was added (Day 0). Scale bars: 500 µm. (B) Experimental schematic for RYU on LONG. (C) Phase-contrast micrograph and immunocytochemistry of RYU with exposure to puromycin. Immunocytochemistry was performed with antibodies to proteins involved in barrier functions (ECAD, CDH17, ZO-1, Claudin-2, Claudin-7) and CDX2. Nuclei were stained with DAPI. Scale bars: 500 (black) and 50 (yellow) µm. (D) Trans-epithelial electrical resistance measurements for RYU. LONG and Caco-2 cells served as negative and positive controls, respectively. White bars are puromycin-untreated RYU on LONG, and black bars are puromycin-treated RYU on LONG. Results are expressed as mean ± SD (n = 3 triplicate biological experiments). Statistical significance was determined using Student's t-test. (E) Lucifer Yellow permeability tests for RYU on LONG. Caco-2 cells served as a control. Black and white bars are treated and non-treated with puromycin, respectively. Results are expressed as mean ± SD (n = 3 triplicate biological experiments). Statistical significance was determined using Student's t-test. *<0.05, **<0.01. (F) Lucifer Yellow permeability test was performed for barrier performance. Barrier performance of RYU (Puromycin-treated (+) and non-treated (-)) was altered in culture. Blue line, Puromycin-treated; orange line, Puromycin-untreated. Results are expressed as mean ± SD (n = 3 triplicate biological experiments). Statistical significance was determined using Student's t-test; *<0.05, **<0.01. (G) Lucifer yellow concentration over time in lucifer yellow permeability tests 16 and 20 days after confluence. Puromycin-untreated (-, orange), Puromycin-treated (+, blue). (H) Phase-contrast photomicrographs of RYU on LONG (Puromycin-treated (+) and non-treated (-)) 16 and 20 days after confluence. Pre- and post-L [file 13287_2023_3629_MOESM7_ESM.pdf]
