# Supplementary material for: Human intestinal organoid-derived PDGFRα + mesenchymal stroma enables proliferation and maintenance of LGR4 + epithelial stem cells
Source: Stem Cell Res Ther. 2024 Jan 17;15:16. doi: 10.1186/s13287-023-03629-5 (PMC10792855; doi:10.1186/s13287-023-03629-5)
Supplement: Supplementary file 4 — Additional file4: Mesenchymal stromal cells promote proliferation of the intestinal epithelial cell organoids (A) Phase-contrast photomicrographs of intestinal epithelial cell (RYU) organoids on mesenchymal stromal cells (LONG) at days 6, 8, and 11 in Matrigel. Left: Intestinal epithelial cell organoids on LONG. Right: Intestinal epithelial cell organoids alone (no feeder cells). Scale bars: 500 µm. (B) Phase-contrast photomicrographs of co-culture of intestinal epithelial cell organoids and LONG on LONG at day 6, 8, and 11 in Matrigel. Left: co-culture on LONG. Right: co-culture alone (no feeder cells). Scale bars: 500 µm. (C) The area of individual intestinal epithelial cell organoids under distinct culture conditions on days 6, 8, and 11. N/A: not available (Organoid boundaries are blurred). Co-culture: RYU and LONG organoids. Feeder: feeder cells (LONG). (D) The area of intestinal epithelial cell organoids under distinct culture conditions on days 6, 8, and 11. Co-culture: RYU and LONG organoids. Feeder: feeder cells (LONG). [file 13287_2023_3629_MOESM4_ESM.pdf]

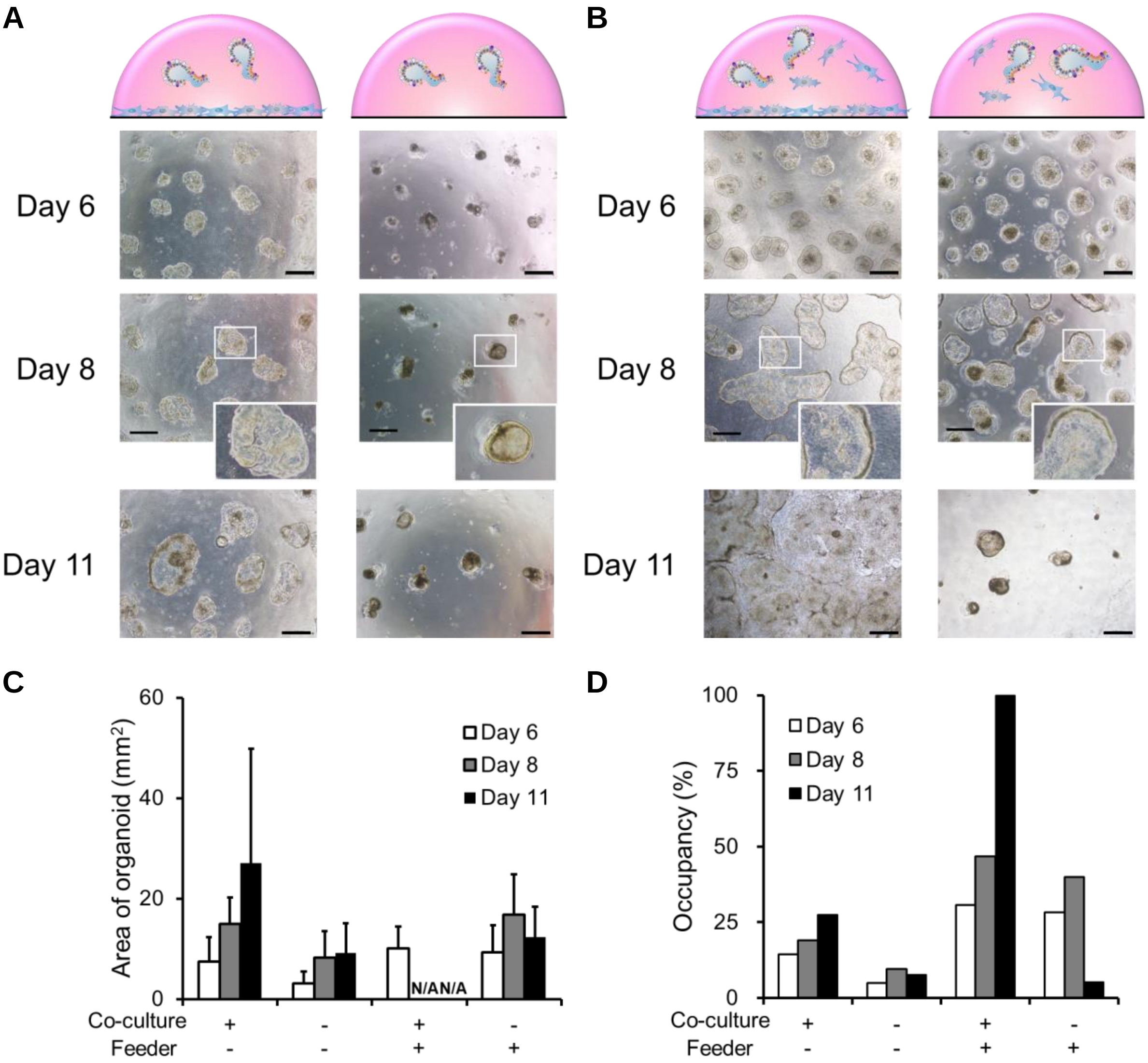

Figure S4. Mesenchymal stromal cells promote proliferation of the intestinal epithelial cell organoids
