# Supplementary material for: Human intestinal organoid-derived PDGFRα + mesenchymal stroma enables proliferation and maintenance of LGR4 + epithelial stem cells
Source: Stem Cell Res Ther. 2024 Jan 17;15:16. doi: 10.1186/s13287-023-03629-5 (PMC10792855; doi:10.1186/s13287-023-03629-5)
Supplement: Supplementary file 5 — Additional file5: Intestinal epithelial cells are positive for crypt base cell (CBC) markers such as LGR4, CD24, and CD44 (A) Immunohistochemistry of RYU in iPGell (upper panel) and human small intestine tissue (lower panel) with antibodies to SOX9. Scale bars: 50 µm. (B) Expression heat map of gene expression in RYU and LONG. n = 3 triplicate biological experiments. (C) Boxplot of representative human intestinal epithelial stem cells and differentiated cell markers. n = 3 triplicate biological experiments (RYU and LONG). Boxplots are expressed as mean ± SD. (D) Immunohistochemistry of RYU and LONG at the subrenal capsule of the kidney 7 days after the implantation (n = 3), using antibodies to CDX2, AE1/3, and Vimentin. Scale bar of zoomed sections represents 100 µm. [file 13287_2023_3629_MOESM5_ESM.pdf]

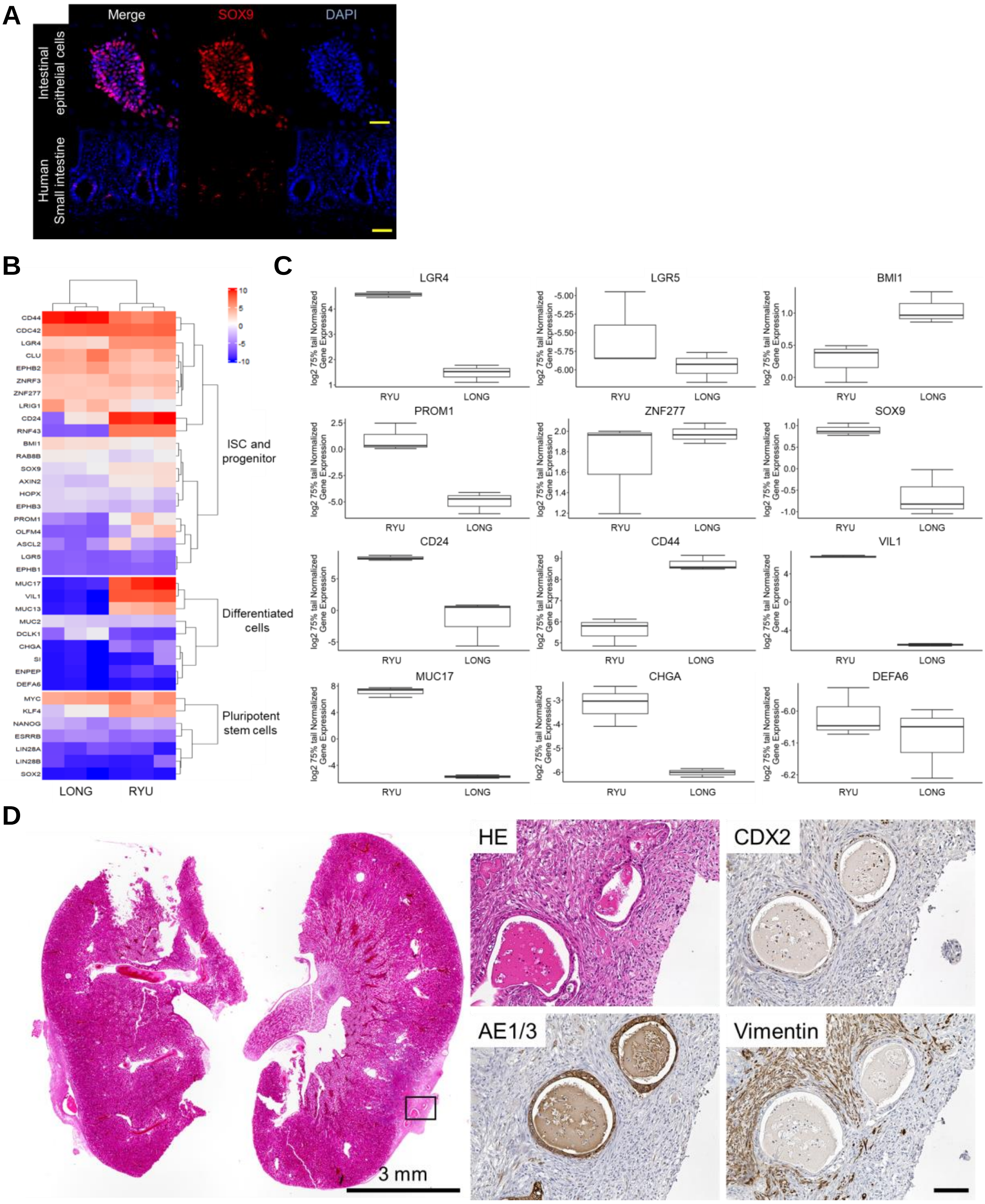

Figure S5. Intestinal epithelial cells are positive for crypt base cell (CBC) markers such as LGR4, CD24, and CD44
